# Sequential Dual Coating with Thermosensitive Polymers for Advanced Fiber Optic Temperature Sensors

**DOI:** 10.3390/s23062898

**Published:** 2023-03-07

**Authors:** Tejaswi Tanaji Salunkhe, Il Tae Kim

**Affiliations:** Department of Chemical and Biological Engineering, Gachon University, Seongnam-si 13120, Gyeonggi-do, Republic of Korea

**Keywords:** dual-polymer-coated sensor, high sensitivity, Fabry–Perot interferometer, thermosensitive polymer

## Abstract

We systematically designed dual polymer Fabry–Perrot interferometer (DPFPI) sensors, which were used to achieve highly sensitive temperature sensors. The designed and fabricated DPFPI has a dual polymer coating layer consisting of thermosensitive poly (methyl methacrylate) (PMMA) and polycarbonate (PC) polymers. Four different DPFPI sensors were developed, in which different coating optical path lengths and the resultant optical properties were generated by the Vernier effect, changing the sequence of the applied polymers and varying the concentration of the coating solutions. The experimental results confirmed that the PC_PMMA_S1 DPFPI sensor delivered a temperature sensitivity of 1238.7 pm °C^−1^, which was approximately 4.4- and 1.4-fold higher than that of the PMMA and PMMA_PC_S1-coated sensor, respectively. Thus, the results reveal that the coating sequence, the compact thickness of the dual polymer layers, and the resultant optical parameters are accountable for achieving sensors with high sensitivity. In the PC_ PMMA-coated sensor, the PMMA outer layer has comparatively better optical properties than the PC, which might produce synergistic effects that create a large wavelength shift with small temperature deviations. Therefore, it is considered that the extensive results with the PC_PMMA_S1 DPFPI sensor validate the efficacy, repeatability, reliability, quick reaction, feasibility, and precision of the temperature readings.

## 1. Introduction

Optical-fiber-based sensors are becoming increasingly mature and in greater demand in the fields of energy, biotechnology, biomedicine, superconducting magnets, automotive technology, aerospace, healthcare, and civil engineering [1,2,3,4,5,6]. These sensors have been extensively researched due to their advantages, such as passive operation, ease of fabrication, compact size, resistance to corrosion, remote sensing ability, capacity for distant sensing, and resistance to electromagnetic interference [7,8,9,10,11]. The two primary categories of optical fiber temperature sensors are optical fiber interferometer and optical fiber gratings. Fiber grating sensors have a low range of temperature measurements and modest sensitivity [12,13,14]. The Fabry–Perot interferometer (FPI) [15,16], Mach–Zehnder interferometer [7], Michelson interferometer [17], and Sagnac interferometer [18] are the four subcategories of optical fiber interferometer temperature sensors. However, high-sensitivity temperature sensors based on FPI have been the focus of recent research. The two most common FPI high-sensitivity temperature sensors are Vernier-effect-based sensors [18,19] and thermal-sensitive material-based sensors. Owing to the drawbacks of Vernier-effect-based sensors, such as their moderate sensitivity, complex structure, and difficulty in manufacturing, research focusing on these sensors is still in progress [18,19].

Thermal-sensitive material-coated sensors have been extensively studied based on the simple Fresnel reflection principle because of their ease of fabrication, repeatability, durability, and low cost. This theory explains the interference problem caused by the discrepancy between the coating material’s thermal expansion coefficient (TEC) and thermo-optic coefficient (TOC). By coating polymers [1,2,11,20,21], carbon nanotubes [22], agarose [23], porous silica xerogels [24], UV-curable resins [25], metal oxides [26], SU-8 photoresistors [12], and metal alloys [26], with affordable and precise cavity dimensions, simple FPI-based temperature sensors were developed. Nevertheless, such single-material/polymer-coated FPI sensors have a significant drawback in that they have limited sensitivity, which is constrained by the thermo-optic properties (i.e., TOC and TEC) of the coating material. To achieve high sensitivity, the FPI configuration should be modified while considering expense, convenience, and repeatability, which are still demanding and challenging. A dual-polymer-coated Fabry–Perot interferometer (DPFPI) sensor is a potential choice to improve the temperature sensitivity [16,27].

Based on the aforementioned inspiration, in this study, we present a systematic study on the fabrication of DPFPI sensors by changing the sequence of the polymer coating and on the optical characteristics and resultant sensitivity. The ferrule connector tip of the single-mode fiber (SMF) is decorated with poly (methyl methacrylate) (PMMA) and a second polycarbonate (PC) via a simple dip-coating method. In addition, sensors coated with the first PC and second by the PMMA polymer are prepared and examined to observe the optical change after changing the sequence of the coating between the two polymers. Moreover, the concentrations of the polymer solutions for coating were varied, and their effects on the sensitivity were studied and compared with those of the corresponding single-polymer-coated sensors. The results reveal that the DPFPI sensors ensure high sensitivity because the dual polymer creates a wide optical path and a significant change in the refractive index (RI), resulting in a large wavelength shift. Six temperature sensors using different coating methods and solution concentrations were fabricated and examined to demonstrate a second-order polynomial fit for temperature sensitivity. Particularly, the PC-PMMA_S1-coated temperature sensor exhibited the best average temperature sensitivity (1238.68 pm °C^−1^) in a 24.4–80 °C temperature range due to the specific sequence of coating, uniform, compact coating, and excellent thermosensitive properties. These results indicate that the sequence of the coating polymers must be considered a crucial parameter to achieve high sensitivity.

## 2. Materials and Methods

### 2.1. Sensing Operation Principle

**Figure 1:**
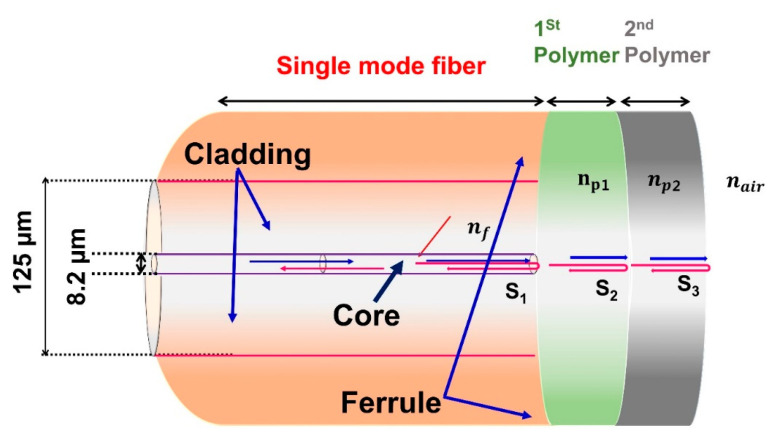
Schematic view of the DPFPI temperature sensor fiber ferrule connector tip.

Figure 1 shows a schematic representation of the ferrule connector fiber-head based on double-polymer layers. DPFPI sensors employ the basic principle of Fresnel reflection, which is an optical phenomenon that occurs when two media with various refractive indices (RIs) come into contact [16,27,28]. In Figure 1, the SMF has the RI *n_f_* = 1.456, where *n_p_*_1_ is the RI of the first polymer and *n_p_*_2_ is the RI of the second polymer coated on the ferrule connector tip of the SMF. The proposed DPFPI sensors have three reflection surfaces: S_1_ is the first surface (SMF/polymer1st), S_2_ is the second surface (polymer 1st/polymer 2nd), and the 3rd surface is S_3_ (polymer 2nd/air), respectively. The Fresnel reflection coefficients for the three reflection surfaces above are R_1_, R_2_, and R_3_, respectively. The three reflected light beams interfere with each other when they return to the SMF because of their distinct optical trajectories. The corresponding reflected light intensities are denoted as S_1_, S_2_, and S_3_, respectively. T_1_ and T_2_ are the relative coating thicknesses of the first and second polymers, respectively. The resultant intensity of the reflected light, determined by the multibeam interference principle, can be stated as follows:

(1) $$\begin{array}{ll} S=S_{1}+S_{2}+S_{3} & -2\sqrt{S_{1}S_{2}}\;cos\left(\frac{4\pi }{\lambda }n_{p1}T_{2}+\varphi_{01}\right)\\ & +2\sqrt{S_{2}S_{3}}\;cos\left(\frac{4\pi }{\lambda }n_{p2}T_{1}+\varphi_{02}\right)\\ & -2\sqrt{S_{1}S_{3}}\;cos\left(\frac{4\pi }{\lambda }\;\left(n_{p1}T_{2}+n_{p2}T_{1}\right)+\varphi_{03}\right) \end{array}$$

The phase factor of the first coated polymer, the second coated polymer, and hybrid cavity (first and second coated polymers) can be expressed in the form of the wavenumber $k=\frac{2\pi }{\lambda }$ while $\varphi_{01}$, $\varphi_{02}$, and $\varphi_{03}$ are the respective initial phases, which can be expressed in Equation (1).

(2) $$\varphi_{1}=k\;\left(2n_{p2}T_{2}\right)+\varphi_{01}\;\varphi_{2}=k\;\left(2n_{p1}T_{1}\right)+\varphi_{02}\;\varphi_{3}=k\;\left(2n_{p2}T_{2}+2n_{p1}T_{1}\;\right)+\varphi_{03}$$

Equation (1) can be expressed in the form of a discrete Fourier transform (DFT) as follows:

(3) $$F\left(\xi_{i}\right)=\sum A_{n}(\xi_{i})\delta \left(\xi_{i}\right).\;$$

The optical paths of the three reflection surfaces are $\xi_{1}=2n_{p2}T_{2}$, $\xi_{2}=2n_{p1}T_{1}$, and $\xi_{3}=\xi_{1}+\xi_{2}$. As a result, the corresponding abscissa values of the peak amplitudes can be used to calculate the thickness of the polymer diaphragms, T_1_ and T_2_, and the RI of the coating materials. Two different approaches were applied for coating, using two thermosensitive polymers with different thermo-optic and thermal expansion coefficients to compare the temperature sensitivity. In this study, PMMA and PC were chosen owing to their good thermo-optic properties, including 1.48, 1.585 for RI, −1.3 × 10^−4^, −0.9 × 10^−4^/°C for TOC, 2.2 × 10^−4^, 1.7 × 10^−4^/°C for TEC, and 80–105, 145 for the glass transition temperature (T_g_) of PMMA and PC, respectively. The sensor was designed with PMMA as the first coating polymer and PC as the second coating polymer, which was compared with the sensor with PC as the first coating polymer and PMMA as the second coating polymer.

The FSRs of the 1st polymer-coated microcavity and the 2nd polymer-coated microcavity are

(4) $$FSR_{1}=\frac{\lambda^{2}}{2n_{p1}T_{1}}\;FSR_{2}=\frac{\lambda^{2}}{2n_{p2}T_{2}}\;\;\;$$

The two microcavities produce the Vernier effect, resulting in the generation of lower-frequency interference spectrum envelope. The FRS of the envelope depends on the 1st polymer- and 2nd polymer-coated micro-cavity, which can be expressed as follow:

(5) $$FSR_{envelope}=\frac{FSR_{1}\times FSR_{2}}{\left[FSR_{1}-FSR_{2}\right]}$$

The temperature sensitivity magnification for the cascaded microcavity structure can be written as

(6) $$M=\frac{FSR_{2}\;}{\left[FSR_{1}-FSR_{2}\right]}$$

During the change in temperature, the 2nd polymer thermally expands, and the refractive index of the polymer is also changed. According to Equation (4), the thickness of the polymer also affects the FSR, and it ultimately affects the sensitivity magnification M. Thus, the interference spectrum shifts, resulting in a change in the interference spectrum envelope of the cascaded DPFPIs. Therefore, the external temperature change can be detected by observing the wavelength shift [29,30,31].

### 2.2. DPFPI Sensor Fabrication and Experimental Set-Up

**Figure 2:**
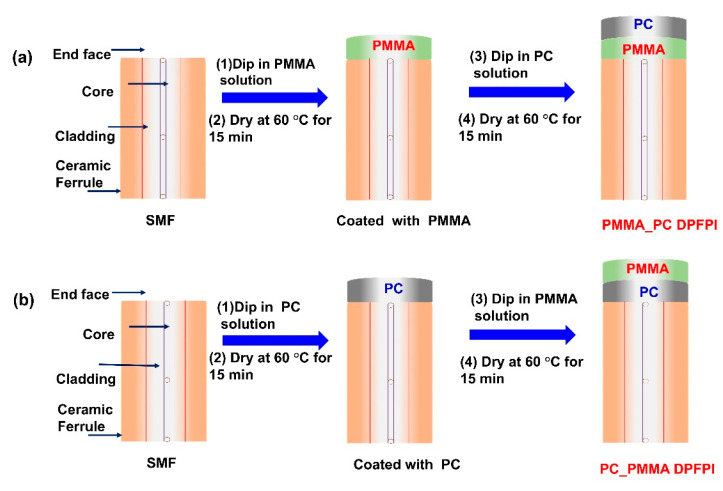
Representation of fabrication method of (**a**) PMMA_PC DPFPI and (**b**) PC_PMMA DPFPI temperature sensors.

The DPFPI temperature sensor’s fabrication process, with simple and stepwise dip coating, is presented in Figure 2. The end tip of the SMF is covered with ceramic ferrule, as shown in Figure S1. Detailed SMF information was provided in our previous study [16]. The selected polymers should have a higher RI than that of the SMF (RI SMF = 1.456) and good adhesive properties with the fiber to make high-quality sensors. In this study, PMMA (Mw~350,000, Sigma-Aldrich, Inc., St. Louis, MI, USA) and PC (goodfellow PE29 6XR England) were selected because of their good properties, as mentioned above. These exceptional properties of the underlying polymers are crucial for creating high-quality temperature sensors [16,20].

DPFPI-based sensors were fabricated using stepwise dip coating, as reported in our previous report [16]. In this study, we varied the concentration of the solution and the sequence of the coating polymers. Solutions of 10 and 15 wt% PMMA/PC in chloroform (≥99.5%, Sigma-Aldrich, Inc., St. Louis, MI, USA) were prepared by simple dissolution with stirring. The ferrule connector SMF tip was cleaned with isopropanol and dried at 25 °C to obtain good polymer adherence to the fiber, which should also be air bubble free. In addition, single-polymer Fabry–Perot interferometer (SPFPI) sensors with PMMA or PC were prepared via the dip coating method in the respective solution for 1 min, followed by oven drying at 60 °C for 15 min, and named as PMMA_SPFPI and PC_SPFPI. During the fabrication of the PMMA_PC DPFPI sensor, the ferrule connector tip of the bare SMF was dipped into a 10 wt% PMMA solution for 1 min and dried in an oven at 60 °C for 15 min. This sensor was used for the next coating after establishing a thin, consistent, smooth, flat, and air-bubble-free PMMA coating. For the second layer of coating, the PMMA-coated SMF was dipped into a 10 wt% PC solution for 1 min and then stored in an oven for 15 min. The resulting sensor was dubbed as a PMMA_PC _S1 DPFPI sensor. The PMMA_PC _S2 temperature sensor was fabricated by the same process, using 15 wt% solutions of the respective polymers. Finally, PC_PMMA S1 (10 wt%) and PC_PMMA_S2 (15 wt%) were fabricated using the same method. However, a PC solution was used for the first coating, and a PMMA solution was utilized for the second coating.

**Figure 3:**
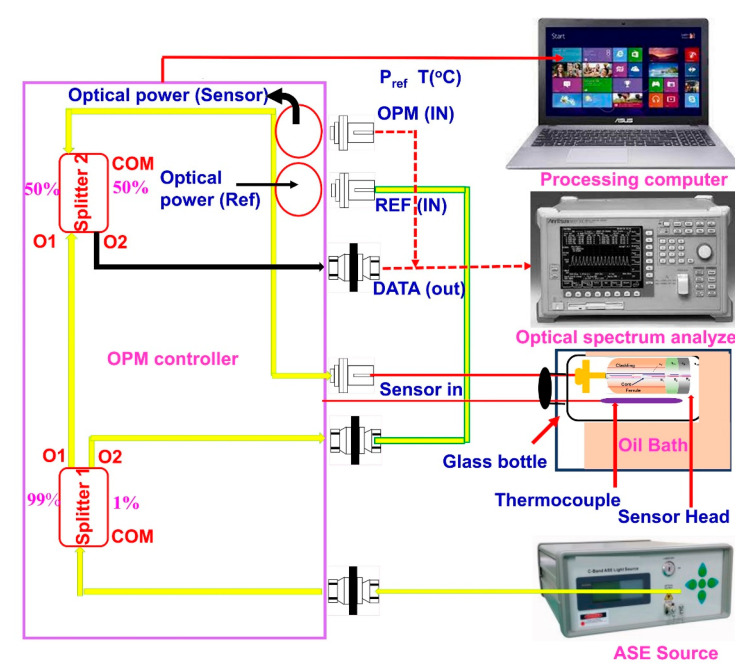
Experimental device setup for measuring the temperature sensitivity of DPFPI sensors.

The temperature response of the fabricated sensor was investigated using the experimental setup shown in Figure 3. The light wavelength emitted by the C-band-amplified spontaneous emission (ASE) broadband source (ASE-BT-C-16-AF) is 1550 nm, which is coupled with the fabricated DPFPI through two coupler optical power controllers (OPCs). The reflection spectra were recorded using an optical spectrum analyzer (OSA) (Anritsu, Kanagawa Prefecture, MA9710C, Japan).

### 2.3. Temperature Response Test

The reflected wavelength shifts of the DPFPI sensors were evaluated with changes in temperature. To regulate the temperature, the ferrule connector tip heads of the DPFPI sensors (GT 307/08 Giltron, New Taipei, Taiwan) and thermocouples with a resolution of ± 0.1 °C were attached together. Then, they were inserted into a glass vial immersed in an oil bath. The ASE-emitted light passed through the OPC and reached the DPFPI sensor. The reflected light was delivered to the OSA, and the OSA showed their responses and displayed the results on the screen. The temperature of the oil bath was increased stepwise from room temperature to 80 °C (137 °C for the PC_SPFPI sensor), and the corresponding wavelength shifts were confirmed by OSA. Subsequently, the system was maintained for natural cooling, and the reflected spectral response was recorded during cooling. An identical test was performed for all the DPFPI sensors, and the average temperature sensitivity was calculated. For the wavelength fluctuation study, we kept the sensors at a constant temperature for 70 min, recorded the wavelength shift results every 5 min, and calculated the standard deviations for the wavelength and temperature.

## 3. Results and Discussion

The FPI sensor with the best temperature sensing quality was obtained when the coating materials had appropriate optical properties in terms of RI, TOC, and TEC. The RI of the selected coating material should be higher than that of silica and air. The RI difference between the coated materials and SMF/air should have considerable values to obtain desirable visibility of the reflected spectra for the FPI sensor [16,28,32]. In this study, we selected PMMA and PC, where the optical properties of these polymers differ from those of silica and air. Therefore, these polymers, including PMMA and PC, could be one of the best choices for FPI sensor applications. Based on Equation (1), the principle of sensing is influenced by the thermal expansion properties and RI change in the coated thermosensitive materials. In Figure 1, the coated ferrule connector tip of the DPFPI sensor functions as a microcavity, and the coated polymer can enlarge or contract as a function of temperature, which supports a change in the RI of the materials. This assists in modifying the optical length of the DPFPI cavity as well as the phase difference between the successively reflected light beams [16,27,28]. An observation of the alteration in interference fringes returned from the three interfaces provides information about the changes in temperature from the ambient condition. This work employed two kinds of sensors: a single-polymer-coated FPI (SPFPI) and a DPFPI sensor. Specifically, we examined temperature sensitivity changes by changing the sequence of coating materials for DPFPI sensors. The proposed PMMA_PC and PC_PMMA DPFPI sensors utilize a simple three-beam interferometric model. On the other hand, the PMMA/PC SPFPI sensors were operated by the simple two-beam interferometric principle. In the DPFPI sensors, the RI change and optical length change were higher with respect to temperature compared to SPFPI sensors.

**Figure 4:**
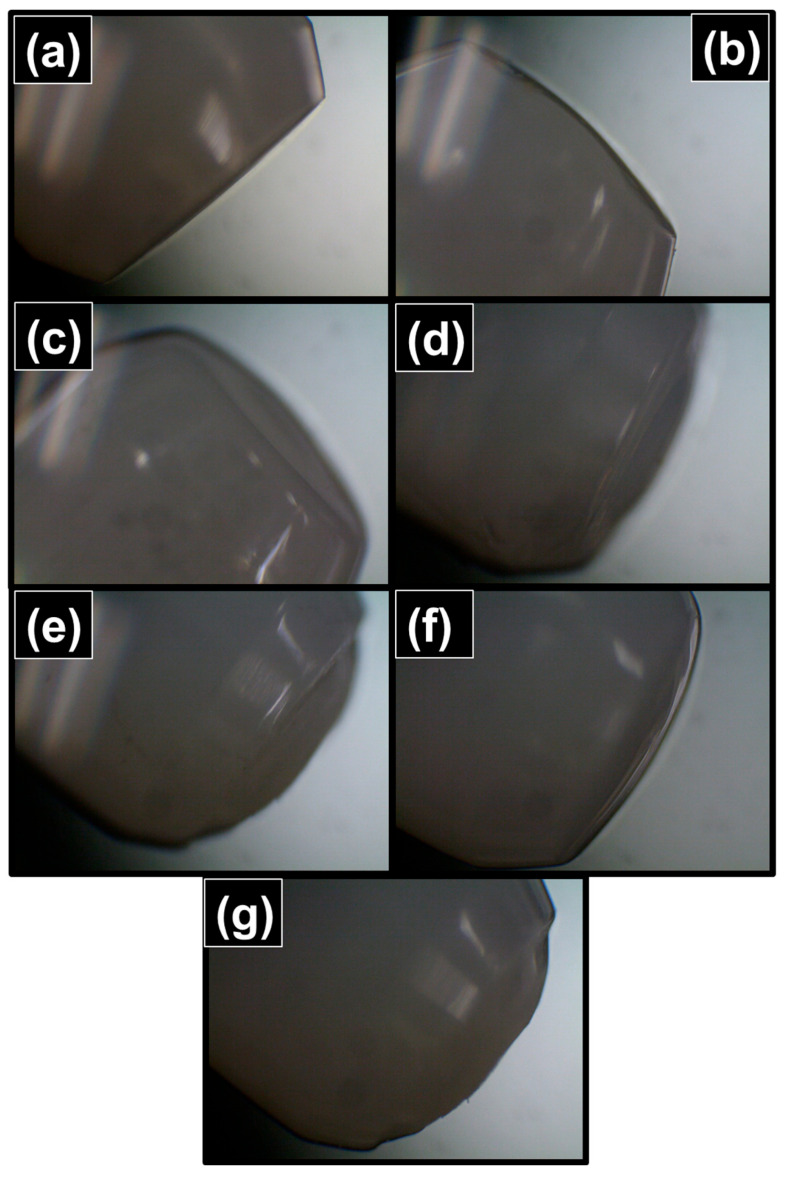
Microscopic view of (**a**) uncoated SMF, (**b**) PMMA_SPFPI, (**c**) PC_SPFPI, (**d**) PMMA_PC-S1, (**e**) PMMA_PC_S2, (**f**) PC_PMMA_S1, and (**g**) PC_PMMA_S2 DPFPI sensors.

**Figure 5:**
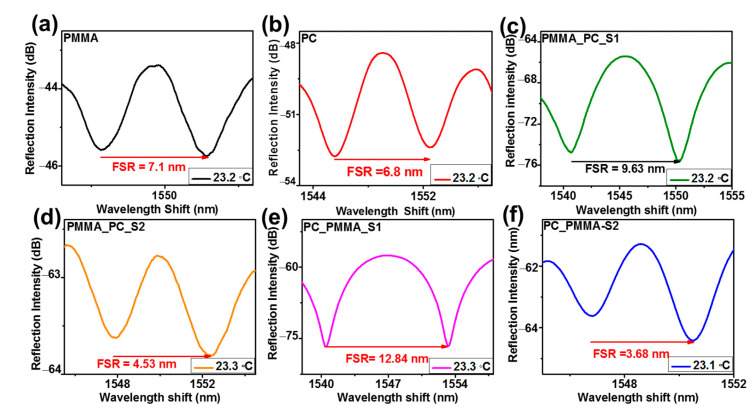
Reflected interference pattern of invented SPFPI (**a**) PMMA and (**b**) PC, DPFPI (**c**) PMMA_PC_S1, (**d**) PMMA_PC_S2, (**e**) PC_PMMA_S1, and (**f**) PC_PMMA_S2.

The coating images of the fabricated sensors were confirmed with optical microscopic images by using INC Microscopes (MIC S16C) (Winona Ave, St. Louis, MI, USA). Figure 4a displays a microscopic view of the standard uncoated SMF, where the ferrule connector tip is clean, spotless, flat, and uniform. A microscopic interpretation of the PMMA and PC SPFPI sensors is illustrated in Figure 4b,c. The coating thickness of the PMMA/PC polymer on the SMF was thin, even, and without air bubbles. The microscopic views of the PMMA_PC-S1, PMMA_PC_S2, PC_PMMA_S1, and PC_PMMA_S2 sensors are shown in Figure 4d–g, respectively. Figure 4d–g prove that DPFPI sensors form thin, clean, flat, and even without bubble coating. Therefore, all microscopic views imply that the adhesive properties of both the polymer with fiber and the polymer with the other polymer are effective in preparing an excellent sensor. If the sensor had irregular, abrasive, and air-bubble-filled coatings, it would reveal undesired reflective spectra [16,20,21]. According to the microscopic view of all the fabricated sensors, they were qualified for temperature sensitivity measurements. The reflected interference patterns of the fabricated sensors were examined at room temperature (Figure 5) to analyze the optical properties of the sensors. The spectra of the reflected beam of the invented sensors (PMMA, PC, PMMA_PC-S1, PMMA_PC_S2, PC_PMMA_S1, and PC_PMMA_S2) are illustrated with 7.1, 6.8, 9.6, 4.5, 12.8, and 6.7 nm free spectral ranges (FSRs) in Figure 5a–f, respectively, at a temperature range of 23.2–23.3 °C. By observing and comparing the spectral response with the microscopic view of the fabricated sensors, the sensors with thick microcavities displayed compact reflected interference spectra, while the compact microcavity sensors reflected rarer interference spectra. Reflected patterns were built based on distinct aspects, such as the method of fabrication, shape, and nature of the coating, RI, and thickness of the microcavity. The reflectivity at each interface and the nature of the polymer coating layer strongly affect the visibility of the resultant reflected interference patterns. The developed PMMA_PC_S2 and PC_PMMA_S2 sensors demonstrate a convex shape in the coating layer (Figure 4e,g) owing to the surface tension of the polymer solution and ferrule connector. As a result, the reflectivity at the polymer air surface is reduced, and the visibility of the resultant spectra was low compared to that with a flat coating layer. The PC_PMMA sensors revealed a good spectral response, implying excellent quality in the interpretation of the intensity of the three reflected interference beams. The three-beam interference pattern that appeared in the PC_PMMA sensors had a stronger reflection intensity at the SMF/PC and PC/PMMA interfaces than that of the PMMA_PC sensor, which could be because the RI difference is higher at interfaces created by the PC_PMMA-type sensor and the thickness of coating. This is also supported by Equations (1) and (4).

**Figure 6:**
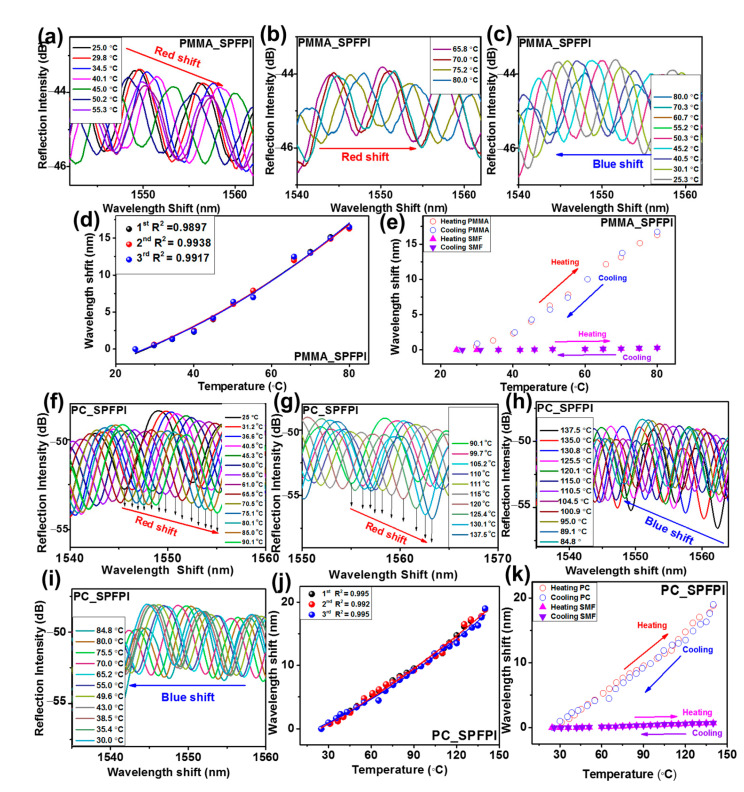
Reflected spectra with increase in temperature (**a**,**b**), with decrease in temperature (**c**) for PMMA_SPFPI, (**f–i**) for PC_SPFPI with increase and decrease in temperature, wavelength shift for three measurements for PMMA_SPFPI (**d**), PC_SPFPI (**j**), average wavelength shift associated with SMF (**e**) for PMMA_SPFPI, and (**k**) for PC_SPFPI sensors.

**Figure 7:**
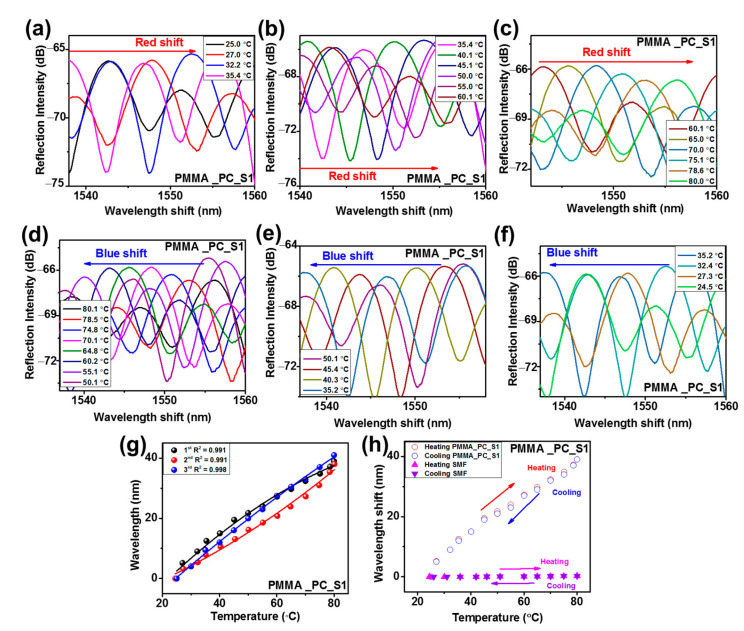
Reflected spectra with increase in temperature (**a**–**c**), with decrease in temperature (**d**–**f**), wavelength shift for three measurements (**g**), and (**h**) average wavelength shift associated with SMF for PMMA_PC_S1 DPFPI.

**Figure 8:**
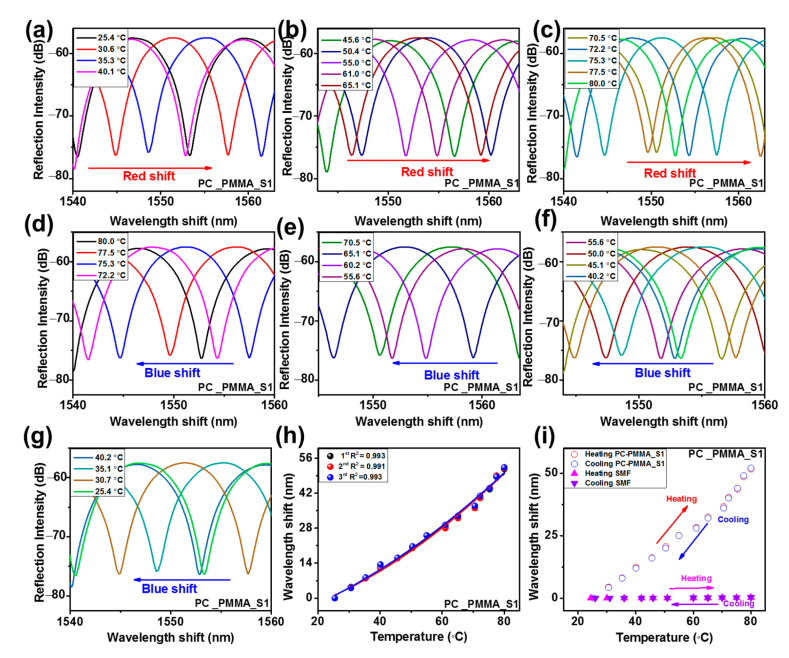
Reflected spectra with increases in temperature (**a**–**c**), decreases in temperature (**d**–**g**), wavelength shift for three measurements (**h**), and (**i**) average wavelength shift associated with SMF for PC_PMMA_S1 DPFPI.

The temperature response performances of the fabricated SPFPI and DPFPI sensors were evaluated, and the obtained results are summarized in Table 1. The reflected interference patterns for the SPFPI and DPFPI sensors illustrated a higher wavelength shift as the temperature increased (Figure 6, Figure 7 and Figure 8, Figures S2 and S4), whereas a lower wavelength shift was detected with cooling (Figure 6, Figure 7 and Figure 8, Figures S3 and S4). Wavelength transformation occurs with a change in temperature because of the change in TEC and the RI change (change in TOC), and two microcavities produce the Vernier effect [7,16,20,21,28]. The PMMA and PC SPFPI sensors delivered an average temperature sensitivity after three measurements of 282.5 and 205.7 pm °C^−1^, respectively, at 25–80 °C and 25–138 °C for the PMMA and the PC SPFPI sensor, respectively. The PMMA SPFPI sensor delivered higher sensitivity than the PC SPFPI sensor because of its considerably higher TEC and TOC. It is noted that if the thickness of the coating increases by increasing the concentration of polymer solution, the sensitivities of the PMMA and PC SPFPI sensors decrease [21]. The PMMA_PC-S1, PMMA_PC_S2, PC_PMMA_S1, and PC_PMMA_S2 DPFPI sensors delivered an average sensitivity after three measurements of 916.7, 654.9, 1238.68, and 751.7 pm °C^−1^, respectively. Notably, DPFPI sensors expressed much higher sensitivity than SPFPI sensors because of the Vernier effect, as mentioned in Equations (4)–(6), which yields comparatively large changes in the interference spectrum with small temperature variations. The comparison of the sensitivity of the PMMA_PC sensor and the PC_PMMA sensor revealed that the PC_PMMA sensor showed higher sensitivity to that of the PMMA_PC sensor. These results illustrate that the coating sequence of the polymer influences the resultant wavelength shift, which could be attributed to the properties of the respective polymers. For instance, the PC_PMMA-coated sensor has a PC inside and a PMMA outside layer coating, with PMMA having higher values of TOC and TEC than PC. As a result, it might have produced synergistic effects to increase the change in the interference spectra. Specifically, this means that the outer layer can expand freely up to its physical limitation (i.e., the higher TOC and TEC of PMMA than PC) because it does not have obstacles to expand; thus, it could show a significant change in the interference spectra at small changes in temperature, and the two microcavities produce the Vernier effect. On the other hand, in the PMMA_PC-coated sensor, PMMA cannot cause a high optical change because of the outer PC coating layer, whereas PC could not cause a higher change in the reflected interference due to its limited TOC and TEC compared to PMMA. Therefore, it was concluded that the obtained results indicate that the coating sequence is a crucial parameter for establishing high-sensitivity and good-quality DPFPI sensors.

The results were examined three times, and the resultant wavelength shifts as a function of temperature are plotted in Figure 6d,j, Figure 7g, Figure 8h, Figures S2g and S4e for PMMA-SPFPI, PC-SPFPI, PMMA_PC-S1, PMMA_PC_S2, PC_PMMA_S1, and PC_PMMA_S2 DPFPI, respectively, to validate the temperature response results, including the stability and repeatability of each sensor. The obtained results illustrate that all sensors could be fitted by the second-order polynomial fitting reversal of the feasibility of the experiments, suggesting that the temperature has a proportional relationship with the wavelength change, with good repeatability and stability of the sensors. The average wavelength shifts over three measurements for each sensor in a range of ~23.1–80 °C for all sensors (except PC, which ranges from 25 to 137.5 °C) are presented in Figure 6e,k, Figure 7h, Figure 8i, Figures S2h and S4f for PMMA-SPFPI, PC-SPFPI, PMMA_PC-S1, PMMA_PC_S2, PC_PMMA_S1, and PC_PMMA_S2 DPFPI sensors, respectively. The second-order polynomial fit with goodness-of-fit coefficients of R^2^ = 0.994, 0.995, 0.998, 1, 0.993, and 0.996 for the PMMA-SPFPI, PC-SPFPI, PMMA_PC-S1, PMMA_PC_S2, PC_PMMA_S1, and PC_PMMA_S2 DPFPI sensors, respectively. Therefore, the developed DPFPI-based sensors showed outstanding stability, reproducibility, and feasibility based on the above results.

**Figure 9:**
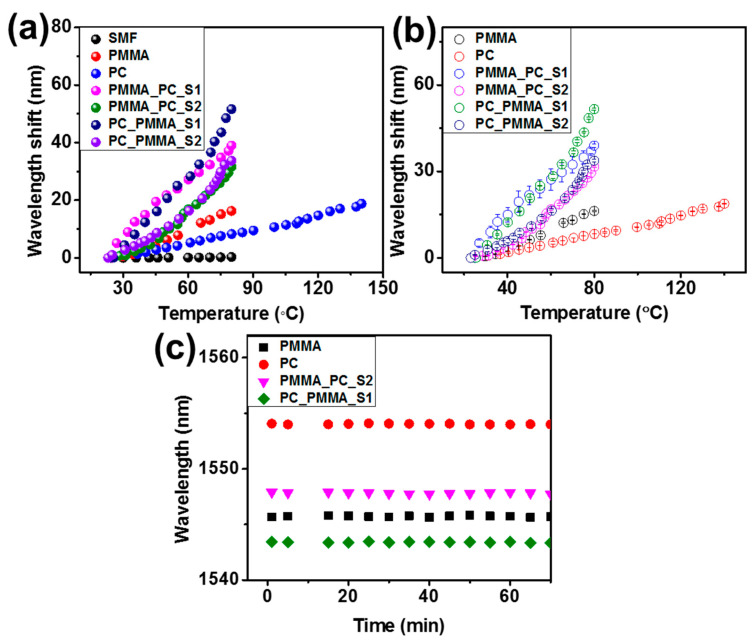
(**a**) Average wavelength shift compared with SMF, (**b**) results of wavelength shift and the standard deviation of the three measurements of PMMA-SPFPI, PC-SPFPI, PMMA_PC-S1, PMMA_PC_S2, PC_PMMA_S1, and PC_PMMA_S2 DPFPI sensors, and (**c**) results of wavelength fluctuation of PMMA-SPFPI, PC-SPFPI, PMMA_PC_S2, and PC_PMMA_S1 DPFPI.

Figure 9a illustrates the results of all sensors. These results were compared with the results of the uncoated SMF. The wavelength shifts with temperature for the plain SMF were not substantial, while the wavelength shifts for the proposed SPFPI were also observed, in which the PMMA_SPFPI sensor shows a much higher wavelength shift than that of the PC-SPFPI sensor because of its higher TOC and TEC values compared to the PC_SPFPI sensor. Meanwhile, the DPFPI sensors delivered significantly higher wavelength shifts than the SPFPI and uncoated SMF sensors. Specifically, the PC_PMMA_S1 (1238.7 pm °C^−1^) sensor demonstrated the best temperature sensitivity, which could be due to the coating length resulting from the concentration of the coating solution along with the synergistic effect, as discussed earlier. The length of the coated polymer is an important parameter for achieving good sensitivity. Sensors with a thin coating length provide high sensitivity, as described in previous studies [16,20,21,27,28]. The small length of the coated polymer could have less restraint to perform volume expansion as well as RI change. Finally, the interference spectral response change with a small temperature change is significantly higher, providing a higher wavelength shift than the thick polymer-coated sensor, as explained in Equations (4)–(6); thickness also affects the spectral response. When comparing to other reported results (Supplementary Materials Table S1) in terms of the sensor preparation as well as the performance, the developed sensors are prepared with a relatively simple preparation method, which is inexpensive, time-saving, and provides better reproducibility and comparable sensitivity[7,16,20,21,27,28,33,34,35,36,37,38].

Figure 9b shows the comparison data of the average wavelength shift, along with their standard error bars. The results indicate that all sensors show precise measurements with good repeatability and feasibility. Specifically, the DPFPI sensors exhibited excellent sensitivity at a high temperature range (~70–80 °C); the results were 1558.9, 902.4, 1820.4, and 1262.6 pm °C^−1^ for PMMA_PC-S1, PMMA_PC_S2, PC_PMMA_S1, and PC_PMMA_S2 sensors, respectively. Finally, the temperature sensors were evaluated to check their stability at a constant temperature (~23.1 °C), and the reflection spectra as a function of time were obtained. The results are presented in Figure 9c and Figure S5, where the standard deviations are 0.0297, 0.023, 0.28, 0.263, 0.076, and 0.079 °C for the PMMA-SPFPI, PC-SPFPI, PMMA_PC-S1, PMMA_PC_S2, PC_PMMA_S1, and PC_PMMA_S2 DPFPI sensors, respectively. The standard deviations in the wavelength shift were 0.73, 0.01, 0.029, 0.057, 0.042, and 0.055 for the PMMA-SPFPI, PC-SPFPI, PMMA_PC-S1, PMMA_PC_S2, PC_PMMA_S1, and PC_PMMA_S2 DPFPI sensors, respectively, indicating great immovability. In addition, the DPFPIS sensor shows a very fast response (within 5–7 s) to the small temperature change (0.3–0.6 °C).

## 4. Conclusions

In summary, DPFPI-based temperature sensors were proposed in a systematic study, where a simple dip-coating method was applied with variation in the sequence of the coating polymers as well as with the concentration change in the polymer solutions. Specifically, the PC_PMMA_S1 DPFPI sensor delivered the highest temperature sensitivity of 1238.7 pm °C^−1^, which was approximately 4.4-, 6-, and 1.4-times higher than PMMA_SPFPI-, PC_SPFPI-, and PMMA_PC_S1-coated sensors, respectively. The excellent performance was attributed to the appropriate sequence for the polymer coating, which significantly altered the reflected interference with a small temperature change resulting from the specific optical properties of the outer layer polymer (PMMA) in the DPFPI sensor. Consequently, a significant spectral shift was observed in the PC_PMMA DPFPI sensor, illustrating that the coating sequence is one of the critical parameters for obtaining highly sensitive temperature sensors. All the fabricated sensors have second-degree polynomial fits that support the feasibility of the experiments, along with good reproducibility, simplicity of fabrication, and low cost. Therefore, the developed PC_PMMA DPFPI sensor with outstanding physical and optical properties could be a viable temperature sensor in various industries.

## Figures and Tables

**Table 1:** Comparative study and results of the SPFPI and DPFPI sensors.

Sensor	FSR (nm)	Average Temperature Sensitivity (pm °C^−1^)	Temperature Sensitivity (pm °C^−1^) (~70–80 °C)	Goodness of Fittine Factor (R^2^)	Standard Deviation for Temperature (°C)	Standard Deviation for Wavelength (nm)
PMMA-SPFPI	7.1	282.5	294.5	0.994	0.0297	0.727
PC_SPFPI	6.8	205.7 (~25–137 °C)	193.7 (~127–137 °C)	0.995	0.0226	0.0098
PMMA_PC_S1	9.6	916.7	1558.9	0.998	0.279	0.029
PMMA_PC_S2	4.5	654.9	902.4	1	0.263	0.0565
PC_PMMA_S1	12.8	1238.7	1820.4	0.993	0.0756	0.042
PC_PMMA_S2	3.7	751.7	1262.6	0.996	0.0785	0.0554

## Data Availability

Not applicable.

## Supplementary Materials

The following supporting information can be downloaded at: https://www.mdpi.com/article/10.3390/s23062898/s1, Figure S1: Pictorial image of SMF with ferrule connector; Figure S2: Red shift reflected spectra of PMMA_PC_S2, and its average wavelength shift; Figure S3: Blue shift reflected spectra of PMMA_PC_S2; Figure S4: Red and blue shift reflected spectra of PC_PMMA_S2 and its average wavelength shift; Figure S5: Wavelength of spectral dip response at a constant temperature and at various time intervals; Table S1: The comparison of optical fiber temperature sensors.
