# Supplementary material for: Sequential Dual Coating with Thermosensitive Polymers for Advanced Fiber Optic Temperature Sensors
Source: Sensors (Basel). 2023 Mar 7;23(6):2898. doi: 10.3390/s23062898 (PMC10059878; doi:10.3390/s23062898)
Supplement: Supplementary file 1 [file sensors-23-02898-s001.zip › sensors-2190036-supplementary.pdf]

# Supporting Information

## Sequential dual coating with thermosensitive polymers for advanced fiber optic temperature sensors

Tejaswi Tanaji Salunkhe[a] and Il Tae Kim[a,*]

[a]*Department of Chemical and Biological Engineering, Gachon University, Seongnam-si, Gyeonggi-do 13120, South Korea*

[*]Corresponding author. Tel.: +82-31-750-8835; Fax: +82-31-750-5363

E-mail addresses: itkim@gachon.ac.kr (I.T. Kim)

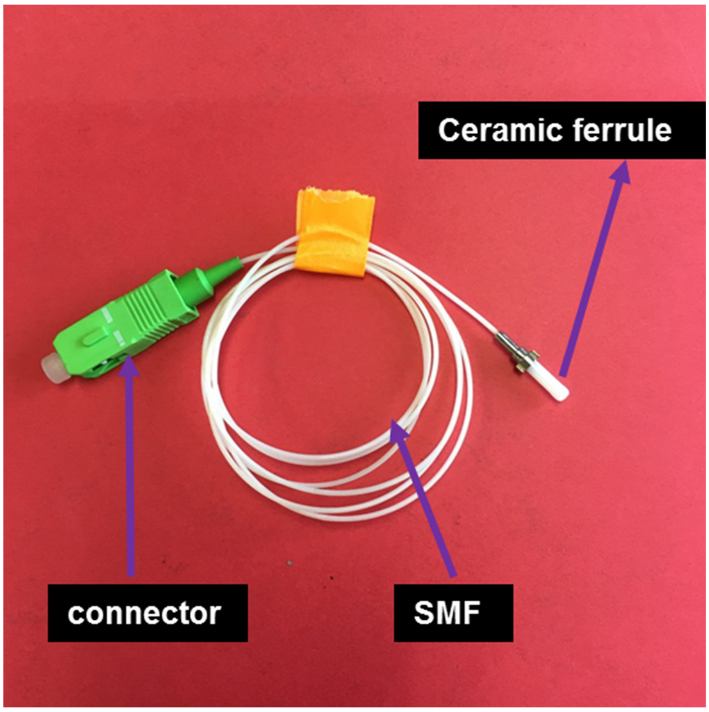


**Figure S1.** The pictorial image of SMF with the ferrule connector.

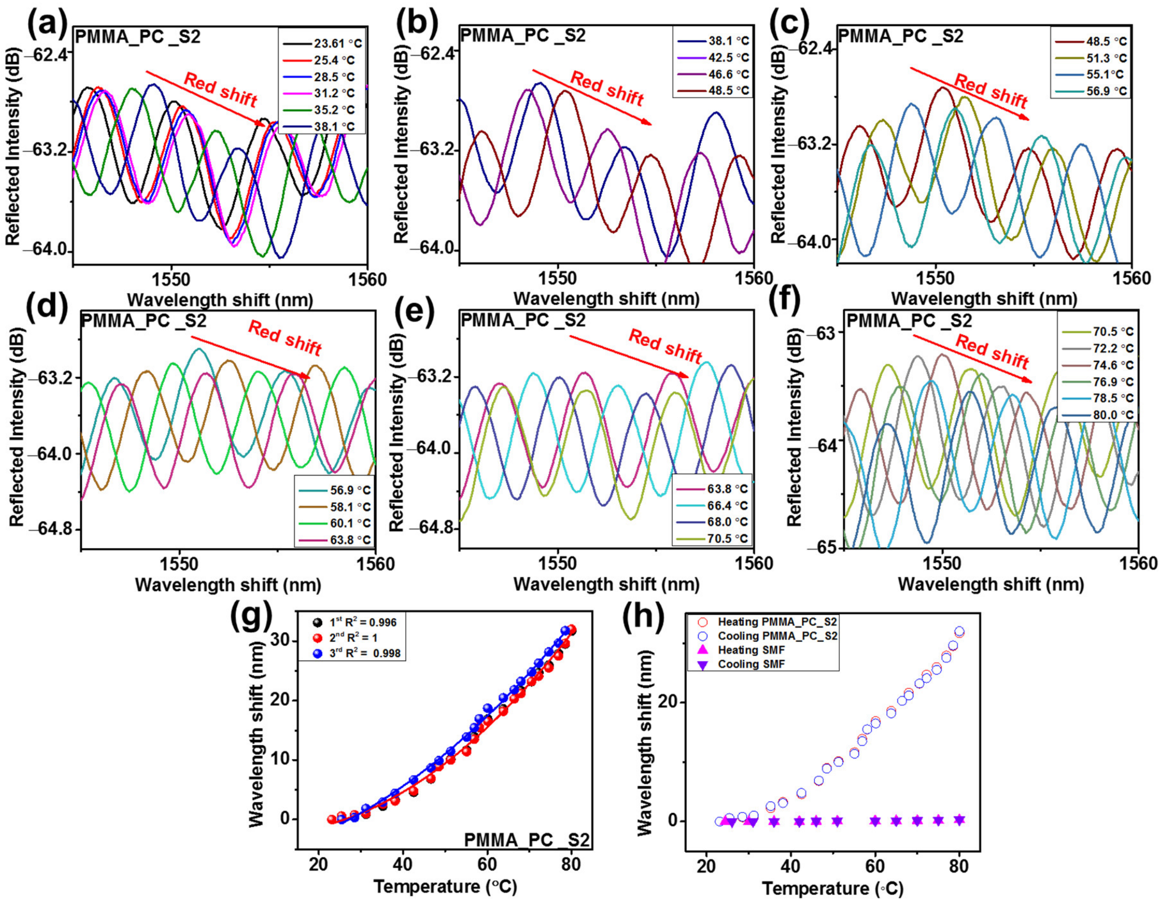


**Figure S2.** Reflected spectra with increase in temperature (a-f), wavelength shift for 3 measurements (g), and (h) average wavelength shift associated with SMF for PMMA_ PC_S2 DPFPI

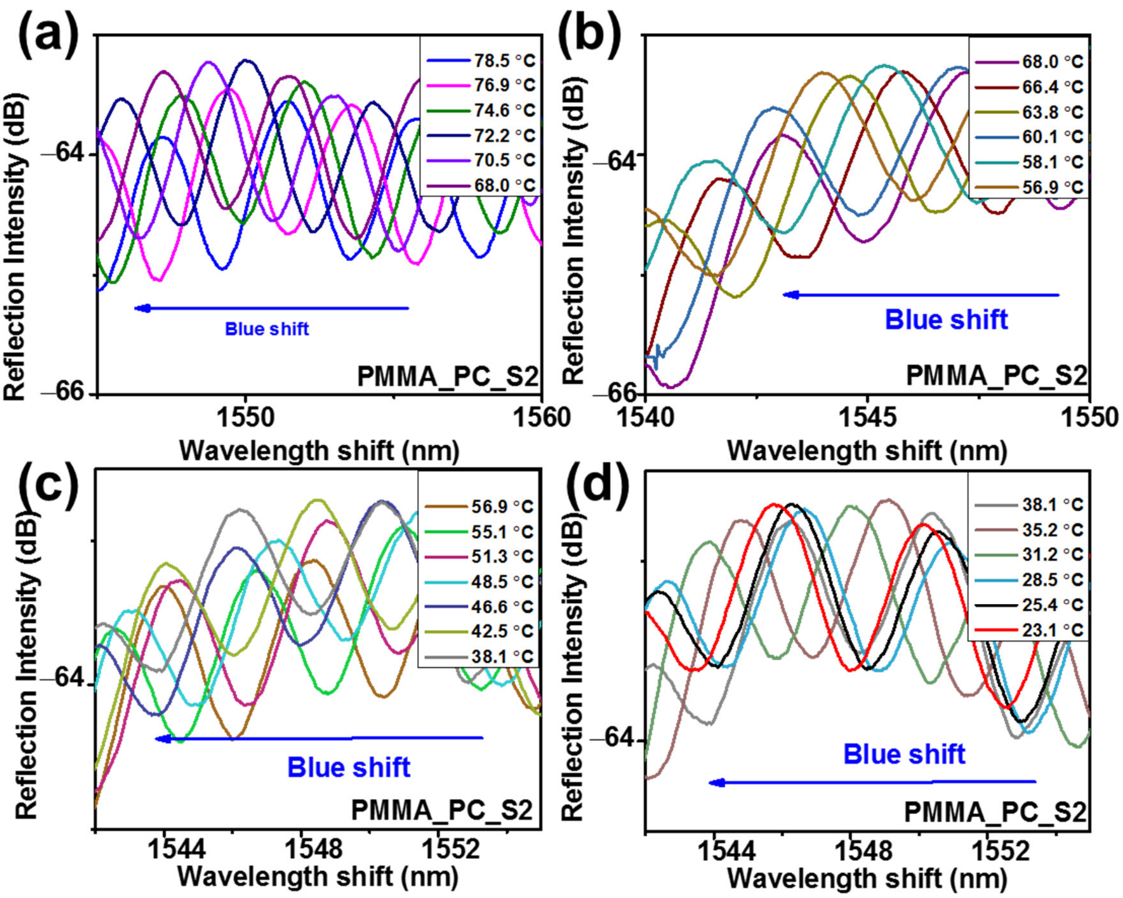


**Figure S3**. Reflected spectra with decrease in temperature for PMMA_ PC_S2 DPFPI

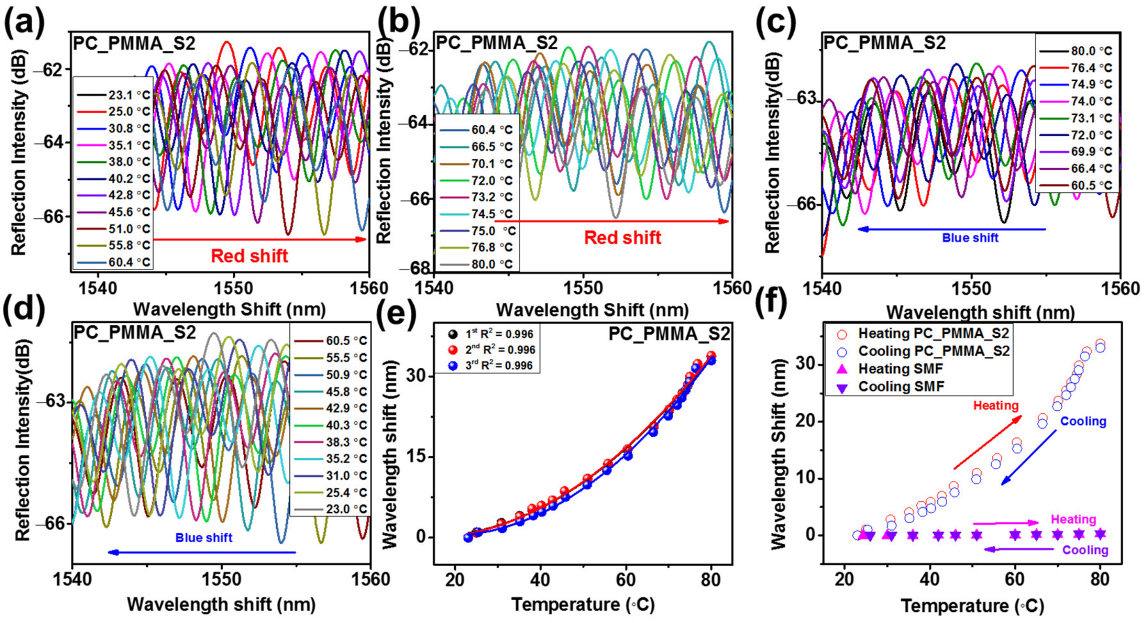


**Figure S4.** Reflected spectra with increase in temperature (a-b), with decrease in temperature (c-d), wavelength shift for 3 measurements (e), and (f) average wavelength shift associated with SMF for PC_PMMA_S2 DPFPI

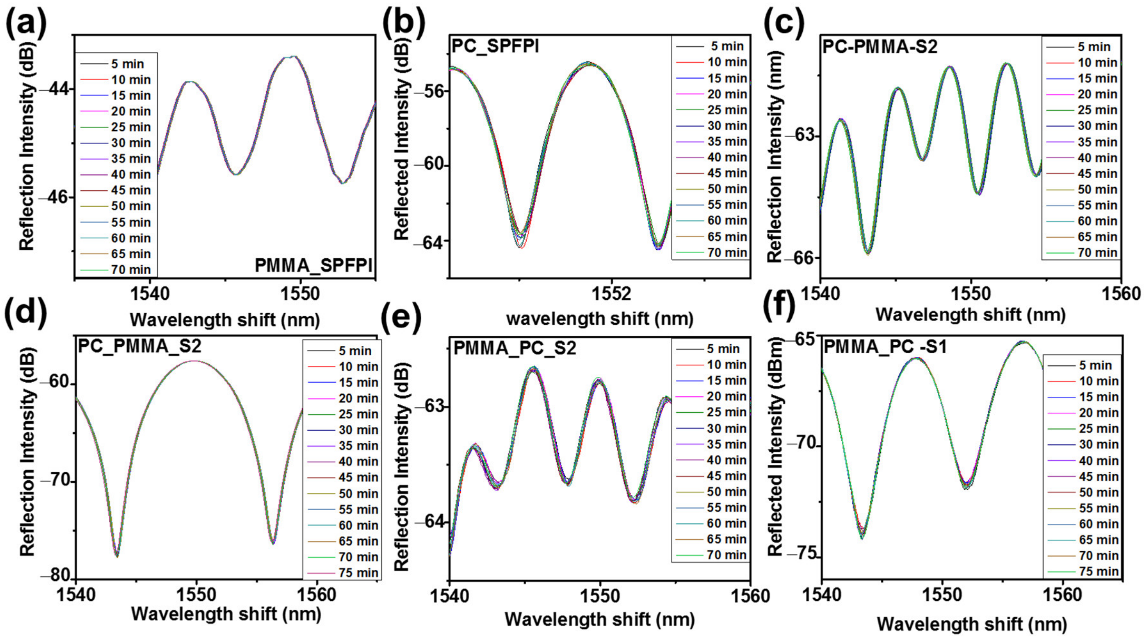


**Figure S5.** Wavelength of spectral dip response at a constant temperature and at various time intervals of (a) PMMA_SPFPI, (b) PC_SPFPI, (c)PC_PMMA_S1, (d)PC_PMMA_S2, (e) PMMA_PC_S1 and PMMA_PC_S2.

**Table S1.** The comparison of optical fiber temperature sensors.

| Type of fiber | Polymer | T(°C) | Sensitivity (pm °C$^{-1}$) | Method | Ref |
|---|---|---|---|---|---|
| Single mode + Hollow core fiber | polydimethylsiloxane | 51-70.5 | 2703.5 | Expensive method, poor reproducibility, requires expensive techniques, complex method | [1] |
| Fabry–Perot Interferometer | Polycarbonate | 20-140 | 245.4 | Low sensitivity, | [2] |
| Microfiber mode interferometer | polydimethylsiloxane | 20-48 | 3101.7 | Requires expensive techniques, | [3] |
| Fabry–Perot Interferometer | Polystyrene | 25-100 | 439.89 | Low sensitivity, | [4] |
| Fabry–Perot Interferometer | step-curing ultraviolet photoresist and polydimethylsiloxane | 20-75 | 689.68 | Low sensitivity, UV curing method needed for coating | [5] |
| Fiber Fizeau interferometer | Norland Optic Adhesive-61 | 10-50 | 269.5 | Narrow operation temperature range, UV curing method needed for coating polymer | [6] |
| Fiber Bragg grating | gold-coated shallow-tapered chirped | 30-80 | 9.893 | $CO_2$ laser splicing system, gold layer sputtering, optical backscatter reflectometer interrogator | [7] |

| | | | | | |
|---|---|---|---|---|---|
| Ultra-long period fiber grating + graded index multimode fiber | doping of germanium | 30-150 | 90.77 | Splicing by arc discharge, fusion splicer | [8] |
| Fiber Bragg grating +Single mode +Multimode Fiber | _ | 0-900 | 13.4 | Femtosecond laser inscription, fusion splicer | [9] |
| Fabry–Perot Interferometer | Poly (vinyl chloride) | 25-60 | 366.0 | Plastic welder used for coating, low sensitivity | [10] |
| Fabry–Perot Interferometer | PMMA_PS | 24.4-80 | 785.5 | Simple dip coating, high sensitivity | [11] |
| Fabry–Perot Interferometer | polyvinyl alcohol | 25-100 | ~193.3 | Low sensitivity, Stepper motor needed for coating | [12] |
| Fabry–Perot Interferometer | PC_PMMA-S1 | 25-80 | 1238.7 | Simple dip coating, high sensitivity reproducibility | **This work** |


[1] M.-q. Chen, Y. Zhao, F. Xia, Y. Peng, R.-j. Tong, High sensitivity temperature sensor based on fiber air-microbubble Fabry-Perot interferometer with PDMS-filled hollow-core fiber, Sensors and Actuators A: Physical, 275 (2018) 60-66.
[2] T. Salunkhe Tejaswi, K. lee Ho, W. Choi Hyung, J. Park Sang, H. Kim Ji, T. Kim Il, High sensitivity temperature sensor based on Fresnel reflection with thermosensitive polymer: control of morphology and coating thickness, Japanese Journal of Applied Physics, 59 (2020) SGGG06-SGGG06.
[3] I. Hernandez-Romano, D. Monzon-Hernandez, C. Moreno-Hernandez, D. Moreno-Hernandez, J. Villatoro,High sensitivity temperature sensor based on Fresnel reflection with thermosensitive polymerr, IEEE Photonics Technology Letters, 27 (2015) 1-1.
[4] Salunkhe, Lee, Choi, Park, I.T. Kim, Enhancing Temperature Sensitivity of the Fabry–Perot Interferometer Sensor with Optimization of the Coating Thickness of Polystyrene, Sensors, 20 (2020) 794.
[5] M. Ge, Y. Li, Y. Han, Z. Xia, Z. Guo, J. Gao, S. Qu, High-sensitivity double-parameter sensor based on the fibre-tip Fabry–Pérot interferometer, Journal of Modern Optics, 64 (2017) 596-600.
[6] C.-L. Lee, Y.-W. You, J.-H. Dai, J.-M. Hsu, J.-S. Horng, Hygroscopic polymer microcavity fiber Fizeau interferometer incorporating a fiber Bragg grating for simultaneously sensing humidity and temperature, Sensors and Actuators B: Chemical, 222 (2016) 339-346.
[7] T. Ayupova, M. Shaimerdenova, D. Tosi, Shallow-Tapered Chirped Fiber Bragg Grating Sensors for Dual Refractive Index and Temperature Sensing, Sensors, 21 (2021).
[8] H. Niu, W. Chen, Y. Liu, X. Jin, X. Li, F. Peng, T. Geng, S. Zhang, W. Sun, Strain, bending, refractive index independent temperature sensor based on a graded index multimode fiber embedded long period fiber grating, Opt Express, 29 (2021) 22922-22930.
[9] X. Sun, L. Zhang, L. Zeng, Y. Hu, J.-a. Duan, Micro-bending sensing based on single-mode fiber

spliced multimode fiber Bragg grating structure, Optics Communications, 505 (2022) 127513.
[10] Z. Zhang, C. Liao, J. Tang, Z. Bai, K. Guo, M. Hou, J. He, Y. Wang, S. Liu, F. Zhang, Y. Wang, High-Sensitivity Gas-Pressure Sensor Based on Fiber-Tip PVC Diaphragm Fabry–Pérot Interferometer, Journal of Lightwave Technology, 35 (2017) 4067-4071.
[11] T.T. Salunkhe, H.K. Lee, H.W. Choi, S.J. Park, I.T. Kim, Simply Fabricated Inexpensive Dual-Polymer-Coated Fabry-Perot Interferometer-Based Temperature Sensors with High Sensitivity, Sensors, 2021.
[12] Q. Rong, H. Sun, X. Qiao, J. Zhang, M. Hu, Z. Feng, A miniature fiber-optic temperature sensor based on a Fabry–Perot interferometer, Journal of Optics, 14 (2012) 045002.
